# 6-﻿[(4-Methylphenyl)sulfonylamino]hexanoic acid: MAK Value Documentation – Translation of the German version from 2023

**DOI:** 10.34865/mb7852139kske10_2or

**Published:** 2025-06-30

**Authors:** Andrea Hartwig, MAK Commission

**Affiliations:** 1 Institute of Applied Biosciences. Department of Food Chemistry and Toxicology. Karlsruhe Institute of Technology (KIT) Adenauerring 20a, Building 50.41 76131 Karlsruhe Germany; 2 Permanent Senate Commission for the Investigation of Health Hazards of Chemical Compounds in the Work Area. Deutsche Forschungsgemeinschaft, Kennedyallee 40, 53175 Bonn, Germany. Further information: Permanent Senate Commission for the Investigation of Health Hazards of Chemical Compounds in the Work Area | DFG

**Keywords:** N-Tosyl-6-aminocapronsäure, Toxizität, Komplexbildner, Entwicklungstoxizität, 6-﻿[(4-methylphenyl)sulfonylamino]hexanoic acid, toxicity, complexing agent, developmental toxicity

## Abstract

The German Senate Commission for the Investigation of Health Hazards of Che﻿m﻿i﻿cal Compounds in the Work Area (MAK Commission) has evaluated the occupation﻿al exposure limit value (maximum concentration at the workplace, MAK value) of 6-﻿[(4-﻿methylphenyl)sulfonylamino]hexanoic acid (*N*-tosyl-6-aminocaproic acid) [78521-39-8] considering all toxicological end points. Relevant studies were identified from a literature search and also unpublished study reports were used. The substance is a complexing agent. As the influence of the complexing effect and the acid effect on the respiratory tract is not known and inhalation studies are not available, a maximum concentration at the workplace (MAK value) cannot be derived. The NOAEL for dev﻿el﻿opmental effects in rats after oral administration was 100 mg/kg body weight and day. In a gavage study in rats that was carried out according to OECD Test Guideline 422, pup mortality was increased and pup body weights were decreased up to postnatal day 4 at 1600 mg/kg body weight and day. The substance is not genotoxic in bacteria or mammalian cells. Carcinogenicity studies are not available. There is no evidence of a contact sensitizing potential. Skin contact is not expected to contribute significantly to systemic toxicity.

**Table:** 

**MAK value**	**not established, see Section II b of the List of MAK and BAT Values**
**Peak limitation**	**–**
	
**Absorption through the skin**	**–**
**Sensitization**	**–**
**Carcinogenicity**	**–**
**Prenatal toxicity**	**–**
**Germ cell mutagenicity**	**–**
	
**BAT value**	**–**
	
Synonyms	6-(((4-methylphenyl)sulfonyl)amino)hexanoic acid
Chemical name (IUPAC)	6-[(4-methylphenyl)sulfonylamino]hexanoic acid
CAS number	78521-39-8
Structural formula	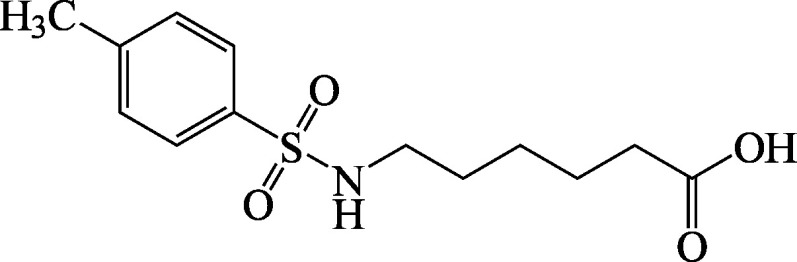
Molecular formula	C_13_H_19_NO_4_S
Molar mass	285.36 g/mol
Melting point	112 °C (ECHA 2021)
Boiling point	substance decomposes at temperatures above 230 °C (ECHA 2021)
Vapour pressure	3.98 × 10^–9^ hPa at 20 °C; 9.11 × 10^–9^ hPa at 25 °C (determined experimentally) (ECHA 2021)
log K_OW_	1.96 at 20 °C (determined experimentally) (ECHA 2021)
Solubility	317 mg/l water at 25 °C (ECHA 2021)
pKa value	4.44 at 20 °C (ECHA 2021)
pH	5.07 (1 g suspended in 100 ml water at pH 6–6.5) (ECHA 2021)
	
Hydrolytic stability	no data
Stability	no data
Production	reaction of 6-aminohexanoic acid with tosyl chloride in an alkaline (pH between 11.3 and 11.7) mixture of water and 1,4-dioxane and acidification (Benacerraf and Levine 1962); reaction of tosyl chloride (dissolved in tetrahydrofuran) with 6-﻿aminohexanoic acid (dissolved in 1 M sodium hydroxide) at pH 9 to 11 (Pavlidis et al. 1988)
Purity	commercial product contains 25% water
Impurities	no data
Uses	as triethanolamine salt used as a corrosion inhibitor in metal working fluids and lubricants (Metall-Chemie GmbH & Co. KG 1999)
Concentrations used	0.2% to 2.5% (Metall-Chemie GmbH & Co. KG 1999)

6-[(4-Methylphenyl)sulfonylamino]hexanoic acid is included in the list of “Komponenten von Kühlschmierstoffen, Hydraulikflüssigkeiten und anderen Schmierstoffen (Components of metal-working fluids, hydraulic fluids and other lubricants)” (Hartwig and MAK Commission 2023, available in German only). It is a derivative of aminocarboxylic acid, which is a complexing agent.

The documentation is based mainly on the publicly available registration data under REACH (ECHA 2021). Cited unpublished toxicological studies from companies have been made available to the Commission.

## 1 Toxic Effects and Mode of Action

In a combined repeated dose toxicity study with reproduction/developmental toxicity screening test carried out according to OECD Test Guideline 422, 6-[(4-methylphenyl)sulfonylamino]hexanoic acid was given per gavage to male and female Wistar rats. At the highest dose tested of 1600 mg/kg body weight and day, the substance caused decreased body weight gains in the males and increased mortality and decreased body weights in the pups on postnatal day 4. There were no unusual findings in the females.

The substance is not irritating to the skin and eyes of rabbits.

6-[(4-Methylphenyl)sulfonylamino]hexanoic acid has not been found to have allergenic effects.

A gavage study in Sprague Dawley rats carried out according to OECD Test Guideline 414 revealed delayed ossification of cranial bones, metacarpals and phalanges in the foetuses and a decreased percentage of males per litter with concomitant maternal toxicity at 400 mg/kg body weight and day and above.

The available studies provide no evidence that 6-[(4-methylphenyl)sulfonylamino]hexanoic acid is mutagenic in bacteria or mammalian cells, or clastogenic in mammalian cells. In vivo genotoxicity, inhalation and carcinogenicity studies are not available.

## 2 Mechanism of Action

There are no data available.

## 3 Toxicokinetics and Metabolism

A toxicokinetics study carried out according to OECD Test Guideline 417 is described only very briefly in the registration dossier. Groups of 4 male and 4 female Wistar rats were given single gavage doses of 6-﻿[(4-methylphenyl)sul﻿fonylamino]hexanoic acid of 800 or 1600 mg/kg body weight with water as the vehicle. Serum concentrations of 6-﻿[(4-﻿methylphenyl)sulfonylamino]hexanoic acid reached their maximum 3 hours after administration and returned to the limit of quantification within 24 hours. Because only urine and faeces samples were collected and metabolites were not analysed, a mass balance is not possible. Due to the sampling conditions, cross-contamination cannot be completely ruled out. Concentrations of 6-[(4-methylphenyl)sulfonylamino]hexanoic acid in the faeces were relatively low. A rel﻿evant amount was excreted with the urine as unchanged substance. The highest concentrations in the faeces and urine were found 6 and 12 hours after administration, respectively, and traces of 6-[(4-methylphenyl)sulfonylamino]hexanoic acid could still be detected up to 168 hours after administration. The authors concluded that 6-[(4-methylphenyl)sul﻿fonylamino]hexanoic acid is readily absorbed after oral administration. The metabolites were not determined in the study (no other details; ECHA 2021). As the metabolism of the substance is not known, it was not radioactively labelled and the metabolites were not investigated, the level of oral absorption is unclear.

There are no data available for the penetration of 6-[(4-methylphenyl)sulfonylamino]hexanoic acid through the skin. Using the mathematical models of Fiserova-Bergerova et al. (1990) and IH SkinPerm (Tibaldi et al. 2014) fluxes of 3.07 and 0.26 µg/cm^2^ and hour, respectively, are calculated, assuming a saturated aqueous solution, a log K_OW_ of 1.96 and a molar mass of 235.86 g/mol. Assuming the exposure of 2000 cm^2^ of skin for 1 hour, this would correspond to absorbed amounts of 6.15 and 0.51 mg, respectively.

In metal-working fluid preparations, 6-[(4-methylphenyl)sulfonylamino]hexanoic acid is used as triethanolamine salt at a maximum concentration of 2.5% (25 g/l) (Hartwig and MAK Commission 2023). For a solution of 25 g/l, the dermal absorption of 485 mg and 40 mg, respectively, is calculated for 6-[(4-methylphenyl)sulfonylamino]hexanoic acid. However, as in these preparations the acid (pKa value: 4.44) is predominantly dissociated, this approach significantly overestimates the amount dermally absorbed.

## 4 Effects in Humans

There are no data available.

## 5 Animal Experiments and in vitro Studies

### 5.1 Acute toxicity

#### 5.1.1 Inhalation

There are no data available.

#### 5.1.2 Oral administration

In a study carried out according to OECD Test Guideline 425 in 5 female Wistar rats, a total dose of 2000 mg 6-[(4-﻿methylphenyl)sulfonylamino]hexanoic acid/kg body weight (purity: 99.24%) was administered by gavage in two fractions during 4 hours (vehicle: water). No signs of systemic toxicity were observed and necropsy did not reveal any abnormal findings. The oral LD_50_ is greater than 2000 mg/kg body weight (ECHA 2021).

#### 5.1.3 Dermal application

In a study carried out according to OECD Test Guideline 402, 5 male and 5 female Wistar rats were exposed occlusively for 24 hours to 2000 mg 6-[(4-methylphenyl)sulfonylamino]hexanoic acid/kg body weight (purity: 99.24%) as a paste in water on shaved dorsal skin. Neither deaths nor irritation at the application site occurred. In addition, no abnormalities were found during the 14-day observation period or at necropsy. The dermal LD_50_ is thus greater than 2000 mg/kg body weight (ECHA 2021).

### 5.2 Subacute, subchronic and chronic toxicity

#### 5.2.1 Inhalation

There are no data available.

#### 5.2.2 Oral administration

A combined repeated dose toxicity study with reproduction/developmental toxicity screening test carried out according to OECD Test Guideline 422 is described in the registration dossier. 6-[(4-Methylphenyl)sulfonylamino]hexanoic acid (purity: 99.24%) was administered by gavage (vehicle: water) to groups of 12 male and 12 female Wistar rats in doses of 0, 100, 400 or 1600 mg/kg body weight and day for 7 days per week. The males were treated for a total of 54 days, 2 weeks prior to mating, during the 2-week mating period, and up to and including 26 days thereafter. The females were treated over a period of 7 weeks, 2 weeks prior to mating, during mating and gestation, and until postnatal day 3. Examination of the females was performed on postnatal day 4. In the high dose group and in the control group, an additional satellite group of 5 animals per sex was included. In male rats, there was a dose-dependent decrease in body weight gains, which was statistically significant at 1600 mg/kg body weight and day, but did not exceed 10%. This effect occurred also in the satellite group, but not in the females. The food intake of the treated animals was not changed compared with that of the control group. According to the information in the registration dossier, a parasite infection may have affected body weights. An osmotic effect of non-absorbed test substance in the intestine is also conceivable. The organs and tissues examined did not reveal any abnormal histological findings. Deaths or adverse clinical findings did not occur. Some statistically significant differences from the control group were found for both organ weights and haematological and clinico-chemical parameters of the treated animals, but they were not related to doses, not confirmed by results from the satellite groups, or were of little biological relevance. The conclusion in the registration dossier is that 6-﻿[(4-﻿methylphenyl)sulfonylamino]hexanoic acid at the highest dose tested of 1600 mg/kg body weight and day led to reduced body weight gains in male rats. A NOAEL (no observed adverse effect level) for systemic toxicity of 400 mg 6-﻿[(4-﻿methylphenyl)sulfonylamino]hexanoic acid/kg body weight and day was derived (see also Section 5.5.1 and 5.5.2) (ECHA 2021). Various data for the administered volume are reported in the registration dossier. According to OECD Test Guideline 422, female animals are treated until at least postnatal day 13. There is no indication why the animals in the study were killed and examined on postnatal day 4. A possible parasite infestation is not plausible, as food intake was not reduced, thus not confirming the supposed lack of appetite. In addition, no effect on the body weight gains of the female animals was found. An osmotic effect is also not likely since the animals did not exhibit diarrhoea.

The original study is not available; therefore, the critical points are not verifiable. The NOAEL for systemic toxicity of 400 mg 6-[(4-methylphenyl)sulfonylamino]hexanoic acid/kg body weight and day is taken from the registration dossier.

#### 5.2.3 Dermal application

There are no data available.

### 5.3 Local effects on skin and mucous membranes

#### 5.3.1 Skin

In a study carried out according to OECD Test Guideline 404, 6-[(4-methylphenyl)sulfonylamino]hexanoic acid (purity not specified) was applied semi-occlusively to the shaved dorsal skin of 3 New Zealand White rabbits (2 males and 1 female). Three gauze patches each with 0.5 g of undiluted test substance were applied to the backs of the animals; exposure was for different lengths of time. For the first animal, the exposure times were 3 minutes, 1 hour and 4 hours. Since no substance-related effects occurred in the first animal, the other two animals were exposed for 4 hours. The animals were examined 1 hour, 24, 48 and 72 hours after removal of the patch. No signs of redness, swelling or other symptoms were observed. The values for oedema and erythema were 0 for all time points. The substance was evaluated as non-irritant to the rabbit skin (ECHA 2021). The test substance was not moistened and made into a paste as recommended in OECD Test Guideline 404 for solids or powders.

#### 5.3.2 Eyes

In a study according to the test guideline EPA OPPTS 870.2400, a commercial product containing around 75% 6-﻿[(4-﻿methylphenyl)sulfonylamino]hexanoic acid and around 25% water (according to the description a white powder) was investigated in the eyes of New Zealand White rabbits (2 males and 1 female). For this purpose, 0.1 cm^3^ of the test substance (91 mg 6-[(4-methylphenyl)sulfonylamino]hexanoic acid) was introduced into the conjunctival sac of one eye of each rabbit. No opacity of the cornea and no iritis were observed after 1 hour and after 24, 48 and 72 hours. After 24 hours, conjunctival irritation was observed in two of three animals (1 × score 0, 1 × score 1, 1 × score 2 of a maximum of 3), which was reversible within 72 hours. The irritation score after 72 hours was 0. Thus, the test substance and its anhydrous form were considered to be non-irritant to the rabbit eye (ECHA 2021).

### 5.4 Allergenic effects

#### 5.4.1 Sensitizing effects on the skin

A maximization test was performed according to OECD Test Guideline 406 in 5 male and 5 female Pirbright Hartley guinea pigs using a commercial product consisting of 75% pure substance and 25% water. Intracutaneous induction was most likely executed with a 5% aqueous solution and topical induction with probably a 50% formulation in petrolatum. None of the animals had produced a reaction 24 and 48 hours after challenge treatment with a 50% formulation in petrolatum (ECHA 2021). The information provided to ECHA regarding the concentrations for induction is contradictory.

Negative results were obtained in a Buehler test carried out according to OECD Test Guideline 406 in 20 male Dunkin Hartley guinea pigs. The topical induction and challenge treatment were performed with a 75% formulation of the substance (purity not specified) in Alembicol D (mainly triglycerides of the C8 and C10 fatty acids of coconut oil). At this concentration, which was the highest possible for the preparation of a formulation, no irritation was observed in a preliminary test. No reactions occurred 24 and 48 hours after the challenge treatment (Huntington Life Sciences Ltd 1999).

#### 5.4.2 Sensitizing effects on the airways

There are no data available.

### 5.5 Reproductive and developmental toxicity

#### 5.5.1 Fertility

In the study according to OECD Test Guideline 422 (see also Section 5.2.2 and 5.5.2), the administration of 6-﻿[(4-﻿methylphenyl)sulfonylamino]hexanoic acid did not cause any effects on the oestrous cycle, histology or the weights of the testes and epididymis. Neither were there any changes in mating and fertility parameters. A NOAEL of 1600 mg/kg body weight and day, the highest dose tested, was derived for effects on fertility (ECHA 2021).

#### 5.5.2 Developmental toxicity

The registration dossier also mentions a study carried out according to OECD Test Guideline 414. In this study, groups of 24 to 25 Sprague Dawley rats were given gavage doses of 0, 100, 400 or 1600 mg 6-[(4-methylphenyl)sulfonylamino]hexanoic acid/kg body weight and day from gestation days 5 to 19 (purity not specified; vehicle: distilled water). At 400 and 1600 mg/kg body weight and day, the dams exhibited decreased body weight gains and decreased gravid uterus weights (no data for statistical significance). The decreased gravid uterus weights correlated with the slightly decreased mean number of foetuses per uterus and increased preimplantation losses. In addition, a statistically significant and dose-dependent decrease (7.3% and 7.9%, respectively) in triiodothyronine levels in the dams at 400 and 1600 mg/kg body weight and day and a statistically significant decrease in thyroxine levels at 1600 mg/kg body weight and day (5.6%) were observed. In the thyroid gland of two dams of this group, minimal degeneration of the follicular epithelium was found in single follicles, which correlated with the decrease in thyroid hormones. In addition, in one female animal, slight C-cell hyperplasia in the thyroid gland was found. Thyroid-stimulating hormone levels and absolute and relative thyroid weights were unaffected by the treatment. In the foetuses, delayed ossification of skull bones, metacarpals and phalanges at and above 400 mg/kg body weight and day were found. At 1600 mg/kg body weight and day, sternoschisis occurred in 1 foetus, which was not observed in the historical control animals. Furthermore, at and above 400 mg/﻿kg body weight and day, the percentage of males per litter was decreased (not statistically significant) compared with that in the control group (400 mg/kg body weight and day: 45.3%; 1600 mg/kg body weight and day: 44.4%), but was lower than in the historical controls (54.1%). In addition, at 1600 mg/kg body weight and day, there was a statistically significant decrease in body weights and a statistically significant increase in anogenital distance in female foetuses; in the male foetuses there were effects on the body weights and anogenital distance. The NOAEL for developmental and maternal toxicity is 100 mg/kg body weight and day (ECHA 2021). The association between the disruption of maternal thyroid homeostasis and delayed ossification in the foetuses discussed by the authors is not verifiable as the original study is not available.

In the study carried out according to OECD Test Guideline 422 (see also Section 5.2.2 and 5.5.1), external examination of the offspring on the day of birth and determination of their body weights did not yield any unusual findings. However, on postnatal day 4, an increased number of dead pups was observed at the high dose of 1600 mg 6-[(4-methylphenyl)sul﻿fonylamino]hexanoic acid/kg body weight and day compared with the number of deaths in the control group. In addition, there were small but statistically significant differences in body weights between the control group and the 400 and 1600 mg/kg groups. Necropsy of the offspring, including those that had died by that time, did not reveal any abnormalities. No toxicity occurred in the dams at the high dose of 1600 mg 6-[(4-methylphenyl)sulfonylamino]hexanoic acid/﻿kg body weight and day; at this level the survival of the offspring was reduced by postnatal day 4. The NOAEL for maternal toxicity was 1600 mg/kg body weight and day, the highest dose tested (ECHA 2021). The NOAEL for perinatal toxicity was 400 mg/kg body weight and day. A study according to OECD Test Guideline 422 does not include a full investigation of teratogenicity.

### 5.6 Genotoxicity

#### 5.6.1 In vitro

In a mutagenicity test carried out according to OECD Test Guideline 471 in the Salmonella typhimurium strains TA97, TA98, TA100, TA102 and TA1535, 6-[(4-methylphenyl)sulfonylamino]hexanoic acid (purity: 99.24%; vehicle: dimethyl sulfoxide) did not lead to increased mutations in the preliminary test at concentrations of up to 5000 µg/plate with and without the addition of a metabolic activation system. In the main study, investigation was performed by both plate incorporation up to 1000 µg/plate and by preincubation up to 500 µg/plate. No cytotoxicity was observed. In a second Salmonella mutagenicity test carried out according to OECD Test Guideline 471, incubation with a 75% 6-﻿[(4-﻿methylphenyl)sulfonylamino]hexanoic acid formulation at concentrations up to 5000 µg/plate in the strains TA98, TA100, TA102, TA1535 and TA1537 with and without the addition of a metabolic activation system did not result in an increase in mutations. Tests were performed using the plate incorporation method and in a second experiment using the preincubation method. Cytotoxicity was observed in the strain TA1537 after preincubation at 5000 µg/plate with and without a metabolic activation system (ECHA 2021). Individual data for mutagenic and cytotoxic end points are not given in the registration dossier.

In a micronucleus test carried out according to OECD Test Guideline 487, 6-[(4-methylphenyl)sulfonylamino]hexanoic acid (purity: 95.57%; vehicle: dimethyl sulfoxide) was not clastogenic either with or without the addition of a metabolic activation system at concentrations up to 875 µg/ml. No cytotoxicity occurred up to 1750 µg/ml (ECHA 2021). It is not clear from the registration dossier in which cell system the test was performed. Both the TK6 cell line and human peripheral blood lymphocytes are mentioned. The number and sex of the blood lymphocyte donors are not provided. According to OECD Test Guideline 487, the use of cytochalasin B is recommended for primary human blood lymphocytes, which is also not mentioned. Also, the preincubation period of 24 hours is too short. Since no individual data are presented, the result cannot be verified.

6-[(4-Methylphenyl)sulfonylamino]hexanoic acid (purity: 99.24%; vehicle: dimethyl sulfoxide) did not produce mutagenic effects in an HPRT (hypoxanthine-guanine phosphoribosyltransferase) assay carried out according to OECD Test Guideline 476 in V79 cells at concentrations up to 5000 µg/ml both with and without the addition of a metabolic activation system. Plating efficiency was reduced by more than 80% at the highest concentration tested of 5000 μg/ml in the absence of a metabolic activation system, and by more than 100% in the presence of a metabolic activation system (no other details) (ECHA 2021). Individual data for mutagenic and cytotoxic end points are not given in the registration dossier.

#### 5.6.2 In vivo

There are no data available.

#### 5.6.3 Summary

The available studies do not provide evidence that 6-[(4-methylphenyl)sulfonylamino]hexanoic acid is mutagenic in bacteria or mammalian cells, or clastogenic in mammalian cells.

### 5.7 Carcinogenicity

There are no data available.

## 6 Manifesto (MAK value/classification)

The most sensitive end points following oral administration of 6-[(4-methylphenyl)sulfonylamino]hexanoic acid are decreased body weight gains in male rats and increased mortality and decreased body weights in the offspring on postnatal day 4. No information on effects in humans is available.

**MAK value and peak limitation. **Inhalation studies are not available. The substance is not irritating to the skin and eyes of rabbits. At a level of solubility in water of 0.3 g/l (ECHA 2021), the substance is neither readily nor poorly soluble.

A combined repeated dose toxicity study with the reproduction/developmental toxicity screening test with 6-﻿[(4-﻿methylphenyl)sulfonylamino]hexanoic acid in male and female Wistar rats is described in the registration dossier. The original study is not available. At the highest dose tested of 1600 mg/kg body weight and day, the substance caused decreased body weight gains in male rats. The NOAEL (no observed adverse effect level) was 400 mg/kg body weight and day. The following toxicokinetic data are taken into consideration for the extrapolation of this NOAEL to a concentration in workplace air: the daily exposure of the animals compared with the 5 days per week exposure at the workplace (7:5), the species-specific correction value for the rat (1:4), the assumed oral absorption (100%), the body weight (70 kg) and the respiratory volume (10 m^3^) of the person, and an assumed 100% absorption by inhalation. The concentration calculated from this is 980 mg/m^3^. The extrapolation of the data from experimental studies with animals to humans (1:2) and taking into consideration chronic exposure (1:4 for the difference in duration between subacute and subchronic studies) results in a concentration of 122.5 mg/m^3^ in the air, from which a MAK value of 100 mg/m^3^ for the inhalable fraction could be derived.

Although a MAK value of 100 mg/m^3^ would protect against systemic effects, it is unclear whether at this relatively high concentration of 6-[(4-methylphenyl)sulfonylamino]hexanoic acid accumulation would occur, with effects on the respiratory tract. The effects of complexation and the pH (acidity) of the substance after inhalation are unknown. As long as there are no inhalation studies available, a MAK value cannot be derived and the substance is therefore assigned to Section II b of the List of MAK and BAT Values. Peak limitation does not apply.

When used in metal-working fluids, adverse effects on the health are not to be expected at the concentration used of 2.5% and provided the technology-based limit of 10 mg/m^3^ is observed, since the maximum concentration is only 0.25 mg/m^3^ and, as the substance is not insoluble, accumulation does not occur.

**Prenatal toxicity. **The registration dossier describes a study carried out according to OECD Test Guideline 414 in Sprague Dawley rats with gavage administration from gestation days 5 to 19. The original study is not available. At 400 and 1600 mg/kg body weight and day, delayed ossification of various bones occurred in the foetuses and there was a decreased percentage of males per litter. At these dose levels, maternal toxicity (decreased body weight gains, decreased gravid uterine weights and evidence of the disruption of thyroid homeostasis) was observed. The NOAEL for developmental and maternal toxicity was 100 mg/kg body weight and day (ECHA 2021). In the study according to OECD Test Guideline 422 mentioned above, the highest dose tested of 1600 mg 6-[(4-methylphenyl)sulfonylamino]hexanoic acid/kg body weight and day caused an increased number of dead offspring on postnatal day 4 and a decrease in body weights which was statistically significant compared with the body weights of the control group. No maternal toxicity occurred at this dose level (ECHA 2021). The NOAEL for perinatal toxicity was 400 mg/kg body weight and day. A study according to OECD Test Guideline 422 does not include a full investigation of teratogenicity.

As no MAK value has been derived, the substance is not assigned to a pregnancy risk group.

**Carcinogenicity and germ cell mutagenicity. **The available studies do not indicate that 6-[(4-methylphenyl)sul﻿fonylamino]hexanoic acid is genotoxic in vitro. Studies of genotoxicity in vivo are not available. Carcinogenic effects due to its structure are not to be expected. Therefore, the substance has not been classified in one of the categories for carcinogens or germ cell mutagens.

**Absorption through the skin. **The NOAEL of 400 mg/kg body weight and day, determined after daily oral administration for 8 weeks in rats, can be used to evaluate the calculated amounts taken up via the skin. Based on the corresponding NAEC (no adverse effect concentration) of 122.5 mg/m^3^ assuming a respiratory volume of 10 m^3^ in 8 hours and 100% absorption by inhalation, a systemically tolerable amount of 1225 mg can be derived for humans. The calculated maximum amount of the substance absorbed through the skin from a saturated aqueous solution under standard conditions (1 hour exposure, 2000 cm^2^ of skin) of 6.5 mg is less than 25% of the systemically tolerable amount. 6-﻿[(4-﻿Methylphenyl)sulfonylamino]hexanoic acid has therefore not been designated with an “H” (for substances which can be absorbed through the skin in toxicologically relevant amounts).

**Sensitization. **There are no findings in humans and no positive results from animal experiments. Therefore, 6-﻿[(4-﻿methylphenyl)sulfonylamino]hexanoic acid has not been designated with “Sh” (for substances which cause sensitization of the skin). As no data for sensitization of the airways are available, 6-[(4-methylphenyl)sulfonylamino]hexanoic acid has not been designated with “Sa” (for substances which cause sensitization of the airways).
